# Perceptions of Resident Autonomy and Decision-Making Opportunities on Pediatric Hospital Medicine Teams

**DOI:** 10.7759/cureus.99459

**Published:** 2025-12-17

**Authors:** Madison Archer, Rena Kasick, Karen Allen, Laura Piper, Nicole Washington, Marquita Genies, Heather Toth, Mohammed Najjar, Michael Weisgerber, Matthew Molloy, Austin Ostermeier, Ndidi Unaka

**Affiliations:** 1 Department of Pediatrics, Division of Hospital Medicine, Cincinnati Children's Hospital Medical Center, Cincinnati, USA; 2 Department of Pediatrics, Division of Hospital Medicine, Nationwide Children’s Hospital, Columbus, USA; 3 Department of Pediatrics, Division of General Pediatrics, Children’s Hospital of Philadelphia, Philadelphia, USA; 4 Department of Pediatrics, Division of Hospital Medicine, Johns Hopkins Children’s Center, Baltimore, USA; 5 Department of Pediatrics, Division of Hospital Medicine, Children's Wisconsin, Milwaukee, WI, USA; 6 Department of Pediatrics, Division of Hospital Medicine, Children's Wisconsin, Milwaukee, USA; 7 Department of Pediatrics, Division of Hospital Medicine, St. Louis Children’s Hospital, St. Louis, USA; 8 Department of Pediatrics, Division of Pediatic Hospital Medicine, Lucile Packard Children's Hospital, Palo Alto, USA

**Keywords:** entrustment, medical education, pediatric hospital medicine, pediatric residency, resident autonomy

## Abstract

Providing autonomy to resident physicians is critical to their professional development. However, opportunities to foster entrustment, and thereby resident-led medical decision making, are often limited in pediatric training. We sought to examine attending physicians' and residents' perceptions of resident autonomy on inpatient pediatric wards with regard to specific medical decisions and patient-care tasks. We conducted a cross-sectional study of pediatric residents and attendings at five academic medical centers in the United States (US): Cincinnati Children’s Hospital Medical Center, Cincinnati, OH; Nationwide Children’s Hospital, Columbus, OH; Children's Hospital of Philadelphia, Philadelphia, PA; Johns Hopkins Children’s Center, Baltimore, MD; and Children’s Wisconsin, Milwaukee, WI. Respondents completed an eight-item survey that rated the percentage of time residents were given autonomy for specific medical decisions. We performed descriptive statistical analysis to compare responses between the groups. Overall, we identified general concordance between attending and resident ratings of autonomy. There were statistically significant differences between resident and attending responses pertaining to antimicrobial choice and addressing patient/family concerns.

## Introduction

Resident trainees require opportunities to practice autonomously and must participate in medical decision-making to progress toward independent practice [1]. Residents who are supported in making autonomous decisions are motivated to take ownership of patient care, pursue their goals, and become high achievers in their field [1-2]. Therefore, residents’ perception of their level of autonomy may impact the development of their medical decision-making skills and self-motivation.

Previous work highlights the frequent discordance in perceptions of resident autonomy between residents and attendings [1-3]. Residents perceive that their autonomy is threatened when attendings provide excessive supervision or when they feel their input is undervalued [1]. Alternatively, the lack of resident experience and motivation, as well as patient safety concerns, contribute to attending hesitancy to grant autonomy [1,4]. This discordance between residents and attendings underscores the need to better understand the areas in which residents perceive they are making autonomous decisions.

In addition, prior literature supports that attendings often struggle to know which medical decisions they can entrust to residents [5]. Less is known about the level of autonomy residents are able to exercise on specific decisions required in their daily care of patients.

The primary objective of our study was to collect baseline data on the amount of perceived autonomy pediatric residents have to make specific medical decisions and complete fundamental physician tasks on inpatient pediatric hospital medicine (PHM) teams. Our secondary objectives included determining if, and to what degree, discrepancies exist between residents’ and attendings’ perceptions of resident autonomy for these decisions and tasks. 

## Materials and methods

We conducted a cross-sectional study of pediatric senior residents and attending physicians on PHM services at five large academic medical centers in the US: Cincinnati Children’s Hospital Medical Center, Cincinnati, OH; Nationwide Children’s Hospital, Columbus, OH; Children's Hospital of Philadelphia, Philadelphia, PA; Johns Hopkins Children’s Center, Baltimore, MD; and Children’s Wisconsin, Milwaukee, WI. In the context of our study, a senior resident is a resident in their second, third, fourth, or fifth year of residency who is responsible for leading the inpatient team under the supervision of an attending and, in some instances, a fellow. All study locations were freestanding children’s hospitals located in the US. These services were staffed by pediatric hospitalists. All programs had pediatric hospital medicine fellows. This work was done concurrently with a quality improvement (QI) initiative at these institutions that aimed to globally increase senior resident autonomy on PHM service. This initiative included interventions such as professional development sessions, expectation-setting huddles, and independent senior-led rounds [6]. This work was approved by the relevant institutional review boards at each participating institution. We sent electronic surveys via email to pediatric senior residents (Appendix A) at the end of their four-week PHM blocks and attendings at the end of their service weeks (Appendix B) from June 2020 to April 2022.

The general framework of the survey was based on a validated survey from Bondi et al. [1] and modified for use in this study. We assessed the respondents’ perception of resident autonomy in seven areas, namely, choosing appropriate antimicrobials, managing intravenous fluids (IVF) and/or enteral feeds, escalating and/or de-escalating respiratory support, ordering a work-up to evaluate diagnostic uncertainty, timing and/or need for specialty consultation, determining discharge readiness, and addressing patient/family concerns. These areas were chosen by group consensus and selected based on their common occurrence on inpatient pediatric hospital medicine teams, based on the experiences of the pediatric hospitalist attendings involved in this study. The respondents were asked to rate how often residents were given the opportunity to make decisions in these areas, with answer choices available on a five-point scale (1 = 0-20% of the time, 2 = 21-40%, 3 = 41-60%, 4 = 61-80%, and 5 = 81-100%). The respondents could select “not applicable” (N/A) if the opportunity to make a particular medical decision did not present itself. In addition, we asked senior residents to rate their general comfort level in making autonomous medical decisions using a five-point Likert-type scale. The respondents were instructed to base their responses on their most recent inpatient PHM service time, four-week blocks for residents, and one-week clinical service time for attendings.

Residents were asked to identify their post-graduate year (PGY); their responses were analyzed both in aggregate and by year of training. The faculty data were analyzed in aggregate. We used Fisher’s exact test (R statistical software, version 3.5.2) to compare survey responses between the following groups: PGY-2 vs. PGY-3, PGY-3 vs. PGY-4+, PGY-2 vs. PGY-3 and PGY-4+, and all residents vs. all faculty. We determined statistical significance using a p-value of < 0.05. Due to the infrequent selection of the 0-20%, 21-40%, and 41-60% responses, we combined these into a single response of <60% for statistical analysis. We removed responses of N/A from the analysis.

The IRB of Cincinnati Children’s Hospital Medical Center determined that the protocol with IRB ID 2020-0411 is considered exempt from full IRB review in accordance with applicable regulations and institutional policy on 06/08/2020.

## Results

During the study period, 209 out of 555 residents (37.6%) responded, and 407 out of 885 attendings (46%) responded. Resident responses included 96 PGY-2s (45.9%), 90 PGY-3s (43%), and 23 PGY-4+s (11%). Of respondents, 547 residents (98.5%) and 881 attendings (99.5%) answered all survey questions. The percentage of total “N/A” responses fell between 0 to 16% depending on the question. 

**Figure 1:**
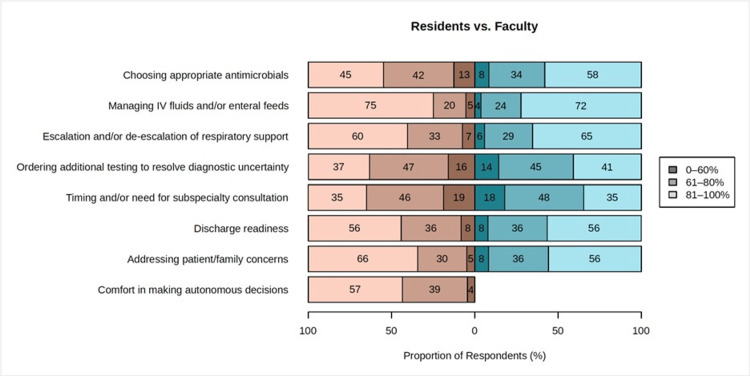
Percentage of perceptions of resident autonomy for residents vs. faculty The left side of the figure represents the resident responses (shades of brown), and the right side represents the faculty responses (shades of blue). The proportion of respondents who feel that residents receive autonomy for each individual clinical decision is represented on the figure in three percentage ranges based on the percentage of time residents are perceived to make the clinical decision autonomously: 0-60%, 60-80%, and 81-100%. All data processing and statistical analysis were performed with the R statistical computing software (version 3.5.2; R Core Team 2018).

More than 90% of all residents and attendings rated senior residents as having autonomous decision-making >60% of the time in the management of IVF/enteral feeds, changes to respiratory support, discharge readiness, and addressing patient/family concerns (Fig. 1). Residents were noted to have the most decision-making opportunities when managing IVF/enteral feeds, with over 70% of resident and attending responses indicating residents had autonomous decision-making opportunities over 80% of the time. Residents were perceived to have the least decision-making opportunities when working up diagnostic uncertainty and deciding timing and/or need for specialty consultation, with less than 34% of residents and less than 41% of attendings reporting that residents had autonomous decision-making opportunities over 80% of the time in these two groups.

Significant differences between resident and attending perceptions of resident autonomy were detected only for antimicrobial choice (p = 0.011) and addressing patient/family concerns (p = 0.046). Attendings rated a higher opportunity for autonomous antimicrobial choice management and a lower opportunity for autonomously addressing patient/family concerns than residents did for themselves. In addition, there were no significant differences in perceptions of autonomous decision-making opportunities between residents based on level of training for any of the surveyed topic areas. Residents did differ in their rating of overall comfort in making autonomous decisions, with PGY-2 residents having less comfort when compared to both PGY-3 and PGY-4+ residents (p = 0.006 and p = 0.008, respectively). There was no significant difference in overall comfort between PGY-3 and PGY-4+ residents (Table 1).

**Table 1:** Differences in perceptions of autonomous decision making between residents and faculty Abbreviations: PGY: post-graduate year; IVF: intravenous fluids; N/A: not applicable a: Areas of perceived autonomy were subdivided into four comparison groups. b: Based on percentage of time that respondents felt residents had autonomy when managing each aspect of patient care. c: P < 0.05 indicates statistical significance.

Areas of perceived autonomy^a^	Differences in the perceptions of autonomy^b^
	P-value^c^
Antimicrobial choice	N/A
PGY-2 vs. PGY-3	0.25
PGY-3 vs. PGY-4+	0.6
PGY-2 vs. PGY-3 & 4+	0.378
All residents vs. all faculty	0.011
Changes in IVF and feeds	N/A
PGY-2 vs. PGY-3	0.121
PGY-3 vs. PGY-4+	0.79
PGY-2 vs. PGY-3 & 4+	0.173
All residents vs. all faculty	0.337
Changes in respiratory support	N/A
PGY-2 vs. PGY-3	0.429
PGY-3 vs. PGY-4+	0.636
PGY-2 vs. PGY-3 & 4+	0.462
All residents vs. all faculty	0.403
Diagnostic workup	N/A
PGY-2 vs. PGY-3	0.72
PGY-3 vs. PGY-4+	0.951
PGY-2 vs. PGY-3 & 4+	0.731
All residents vs. all faculty	0.608
Need for consults	N/A
PGY-2 vs. PGY-3	0.706
PGY-3 vs. PGY-4+	0.605
PGY-2 vs. PGY-3 & 4+	0.839
All residents vs. all faculty	0.935
Determining discharge readiness	N/A
PGY-2 vs. PGY-3	0.813
PGY-3 vs. PGY-4+	0.589
PGY-2 vs. PGY-3 & 4+	0.953
All residents vs. all faculty	0.973
Addressing patient concerns	N/A
PGY-2 vs. PGY-3	0.23
PGY-3 vs. PGY-4+	0.339
PGY-2 vs. PGY-3 & 4+	0.251
All residents vs. all faculty	0.046
Comfort with autonomous decisions	N/A
PGY-2 vs. PGY-3	0.006
PGY-3 vs. PGY-4+	0.135
PGY-2 vs. PGY-3 & 4+	0.008
All residents vs. all faculty	N/A

## Discussion

To our knowledge, this is the first paper describing the perceptions of autonomy for specific medical decisions that pediatric residents make in inpatient hospital medicine settings. This study sheds light on the intricacies of resident medical decision-making on pediatric inpatient wards and highlights opportunities to foster entrustment and encourage resident autonomy. Interestingly, we found that the majority of residents and attendings perceived that autonomous decision-making opportunities were granted to residents most of the time in all categories. Furthermore, attending and resident perceptions of autonomy regarding specific medical decisions were largely concordant, except for in the management of antimicrobials and addressing patient/family concerns. Areas in which autonomy is perceived as lower or where there is discordance between attending and resident perceptions may provide opportunities for attendings on the inpatient wards and residency program directors to identify specific areas for improvement. Curricular adjustments for targeted interventions, specifically in areas of clinical reasoning and in strategies to mitigate diagnostic uncertainty, may help attendings and residents feel more comfortable increasing autonomy.

This study showed a higher concordance between attendings’ and residents’ perceptions of autonomy than what has been previously reported in the literature [1,3,4,7]. A possible explanation for this finding is that most previously published work has described a more general view of autonomy [1,4,7], while our work focused on specific decisions made in daily inpatient pediatric practice. Our approach, using more specific survey questions, may have allowed for increased objectivity in responses.

Residents and attendings agreed regarding the decisions in which residents had the least autonomy: consulting subspecialists and ordering additional diagnostic testing. Given the well-documented contribution of diagnostic uncertainty to diagnostic and medical errors [8], it is likely that attendings desire to give additional oversight when diagnostic uncertainty is present. Responses were discordant in two areas: antibiotic choice and addressing patient/family concerns. We identified several contributing factors that may explain these findings. One study site was involved in QI work to increase the timely transition of patients from parenteral to enteral antibiotics. This may have contributed to resident perceptions of less autonomy in this category. In addition, attendings may override resident decisions more frequently for the selection of appropriate antibiotics, given that management is less flexible or subjective than decisions in other domains, like for the management of IVF or enteral feeds.

Attending perceptions of residents having less autonomy in addressing patient/family concerns likely stem from several factors. While attendings typically directly observe residents communicating with families on rounds, they are often not present when residents communicate with families at other time points throughout the day and night. Hence, sparse observations of residents addressing family concerns may result in attending uncertainty about how residents navigate conversations with families. The reason for the discordance in perceptions of resident autonomy in these two categories is an area that could be explored in future work. Lastly, we found PGY-2 residents cited less comfort making autonomous decisions than more experienced PGY-3 and PGY-4+ residents. This is unsurprising given previous studies show that opportunities to make autonomous decisions in addition to experience over time are critical to building resident confidence [1].

Our study should be interpreted in the context of some limitations. While this study was a multicenter study, all sites were large academic hospitals with dedicated PHM teams and attendings. The findings may not be generalizable to smaller hospitals and residency programs with different inpatient team structures. Second, the results of the study may be impacted by recall bias, given likely time lags between respondents completing time on a PHM service and filling out the surveys, and social desirability bias, given respondents' knowledge that higher resident autonomy is a desirable outcome [9]. Response bias may also be present, given the lower response rate for our surveys. Third, survey responses were not studied over time, and the amount of autonomy granted likely differs between inexperienced senior residents compared to more experienced senior residents later in the academic year. In addition, though the QI interventions implemented by our study group did not target specific medical decision-making opportunities, they likely had an impact on general perceptions of autonomy. It is possible that the ongoing QI work led to more congruence between residents and attendings regarding perceived autonomous decisions than prior studies. The congruence observed may also be due to the narrow scope of the survey questions. Fourth, although autonomy is a common concept within medical education, we did not explicitly define this term for survey respondents. Nuanced differences in the interpretation of what autonomy means may exist at the institutional and individual level that could have affected responses and overall measurement validity. Despite limitations, this work serves as a starting point to further explore perceptions of resident autonomy for specific clinical decisions and the effect those perceptions may have on actual autonomy.

## Conclusions

This study explored the degree of perceived resident autonomy for specific medical decisions on general pediatrics inpatient wards. Pediatric residents led the decision-making the majority of the time for medical decisions with a broader range of acceptable approaches, while diagnostic uncertainty hindered the degree of resident autonomy. Residents and attendings had discordant perceptions of resident autonomy for less flexible medical decisions, such as antimicrobial selection. Educational interventions to foster resident clinical reasoning and mitigate diagnostic uncertainty may help to bolster opportunities for resident-led medical decision-making. Further exploration of strategies to promote resident entrustment will additionally support the development of autonomous practice during pediatric residency training.

## Figures and Tables

**Figure 2:**
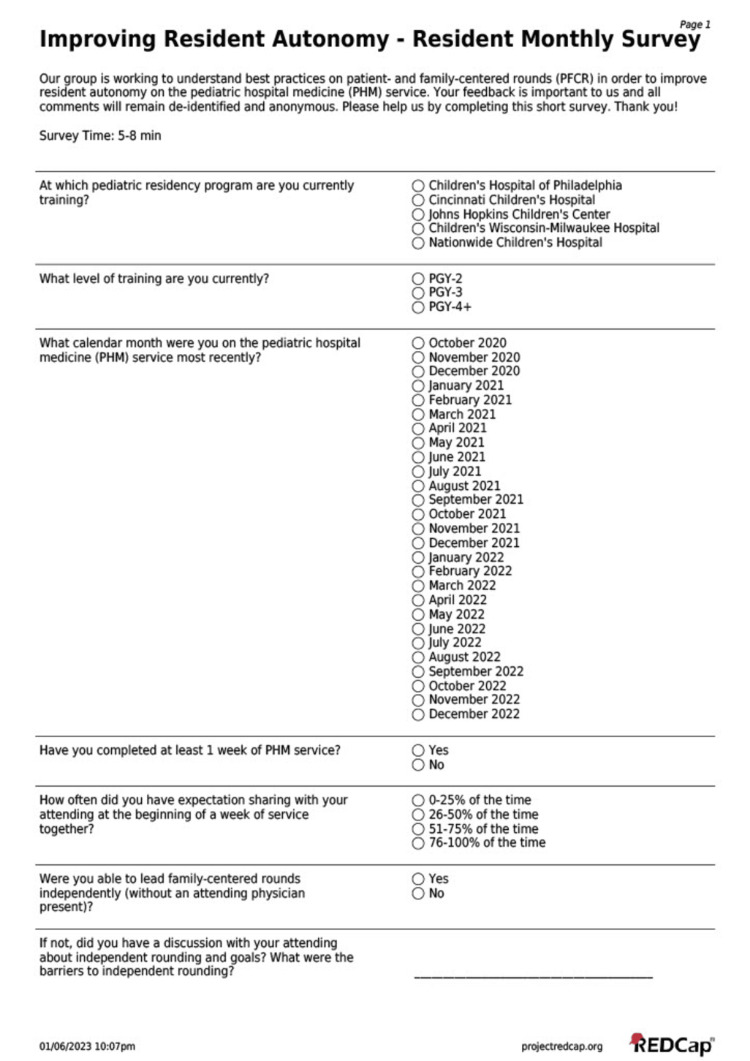
Resident Monthly Survey page 1

**Figure 3:**
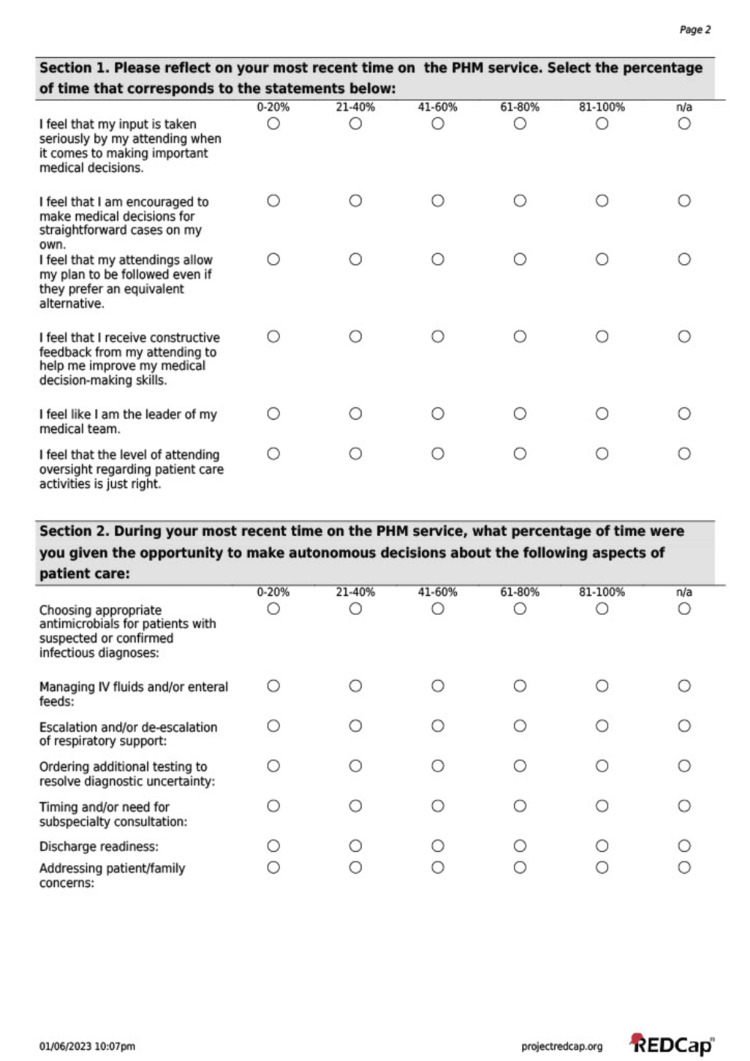
Resident Monthly Survey page 2

**Figure 4:**
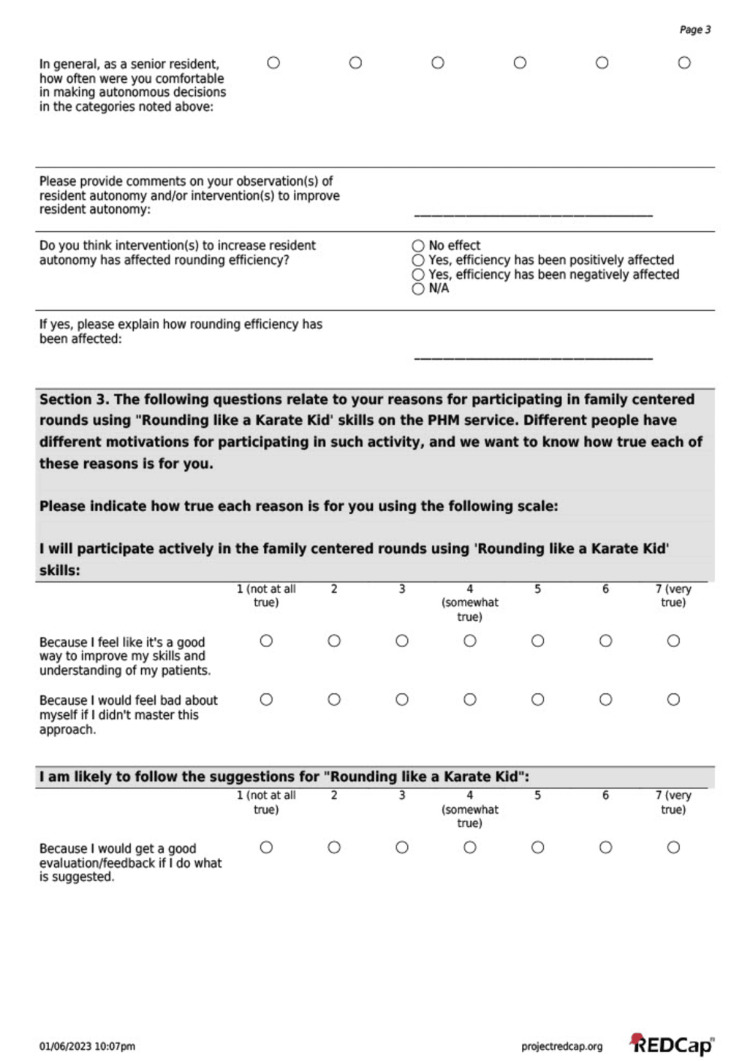
Resident Monthly Survey page 3

**Figure 5:**
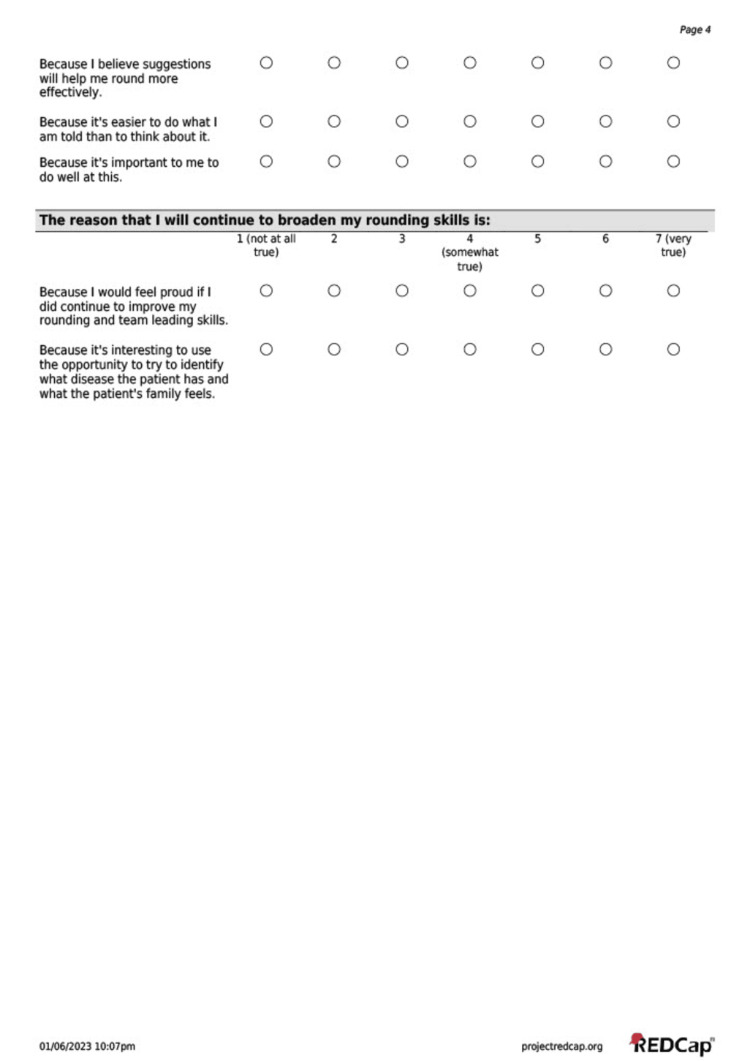
Resident Monthly Survey page 4

**Figure 6:**
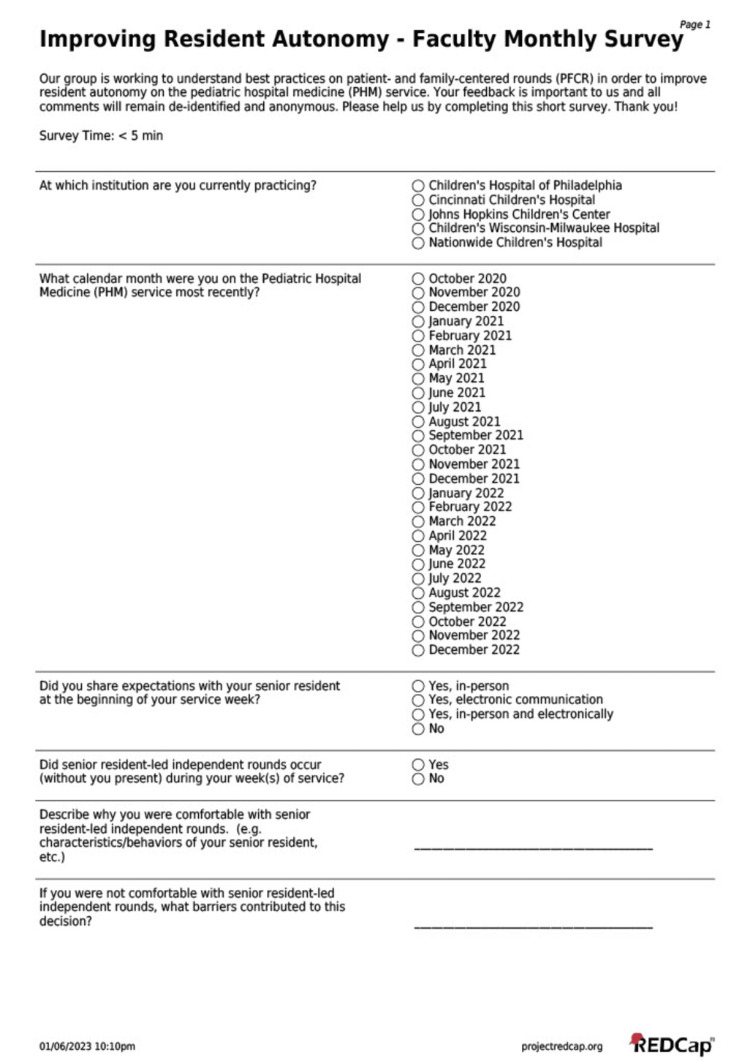
Faculty Monthly Survey page 1

**Figure 7:**
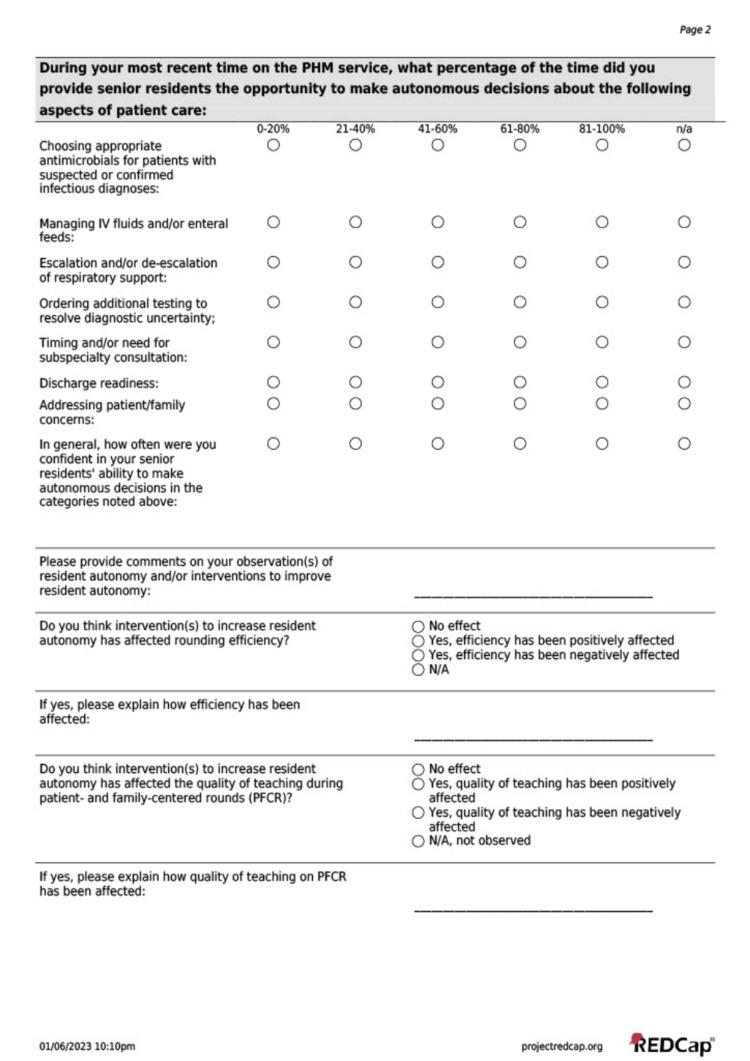
Faculty Monthly Survey page 2

**Figure 8:**
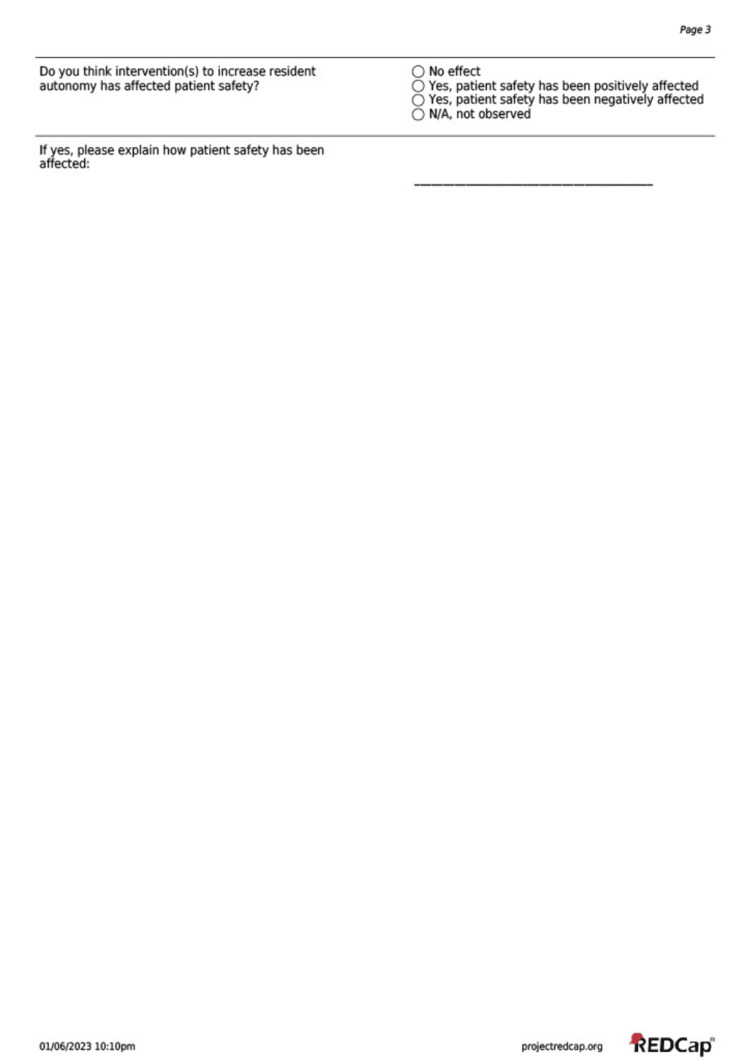
Faculty Monthly Survey page 3

## Appendices

Appendix A

Resident Monthly Survey

Appendix B

Faculty Monthly Survey
